# Insights and achievements from the Tara Pacific expedition

**DOI:** 10.1038/s41467-023-38896-6

**Published:** 2023-06-01

**Authors:** Serge Planes, Denis Allemand

**Affiliations:** 1 Laboratoire d’Excellence “CORAIL,” PSL Research University: EPHE-UPVD-CNRS, UAR 3278 CRIOBE, Université de Perpignan, Perpignan Cedex, France; 2 Centre Scientifique de Monaco, 8 Quai Antoine Ier, MC-98000 Monaco, Principality of Monaco

**Keywords:** Ecological genetics, Marine biology

## Abstract

The Tara Pacific program and expedition focused on coral reefs across the Pacific Ocean and used a coordinated sampling effort to address questions at multiple scales using a common suite of samples. Here, we highlight some of the Tara Pacific achievements, discussing the benefits of long-duration sea expeditions for investigating a wide array of research questions within a selected ecosystem.

Coral reefs are the most biologically diverse marine ecosystem on the planet, hosting about 5% of the world’s known species and about 35% of known marine species in a surface area of only 0.16% of the ocean^1^. The Pacific Ocean, including the South Asian Sea, contains about 60% of the world’s coral reefs, hosting many important biodiversity gradients as well as the most biodiverse region in the oceans, the coral triangle including Indonesia, Philippines, and Papua New Guinea.

Based on a 2-year-long continuous scientific expedition at sea, which traversed the coral reefs of the Pacific Ocean and the Asian seas, the Tara vessel covered a journey of more than 100,000 km with about 3000 scuba-dives (Fig. 1). Similar to earlier long expeditions like those of James Cook in the 18th century, Charles Darwin on the Beagle (1831–1836), or James Dana (1838–1842) which helped develop theories on reef formation and reef biodiversity (see^2^ for a history of reef research), the Tara Pacific expedition aimed to provide a baseline of the ‘omics’ complexity of the coral holobiont (the Cnidarian animal host and its resident microbial assemblage) and its ecosystem across the Pacific Ocean^3^.

**Fig. 1:**
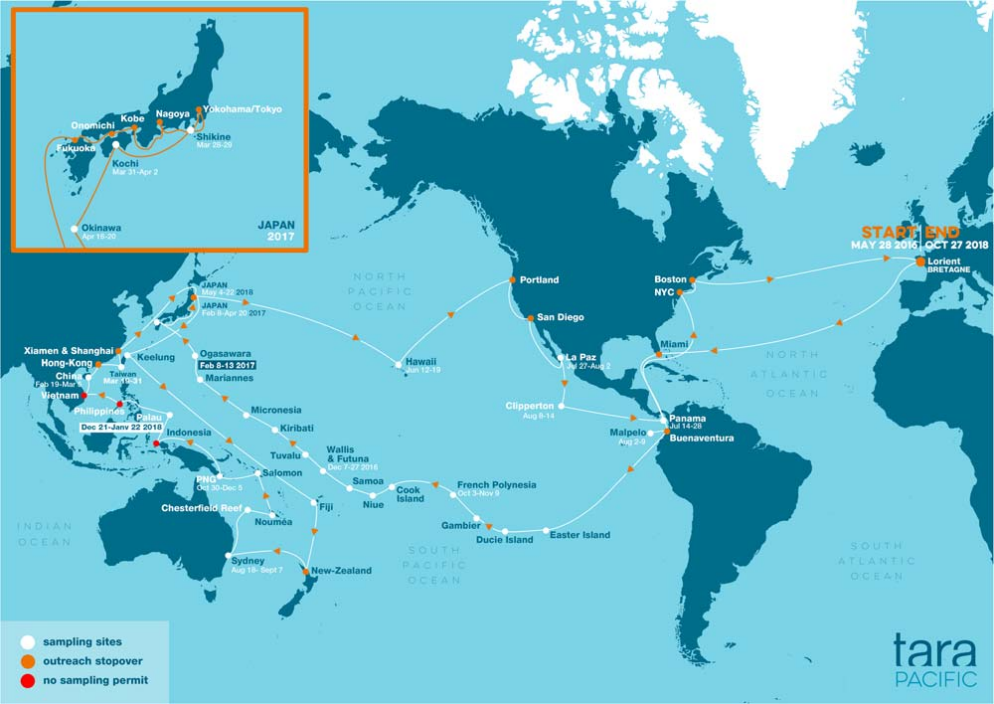
Tara Pacific expedition map (2016–2018) with sampled coral systems (white circles). Reproduced with permission from The Tara Ocean Foundation.

The Tara Pacific program used very high-throughput genetic sequencing and molecular analyses to reveal the entire microbial and chemical diversity of coral holobionts as well as their functional traits at a basin-wide scale. This ambitious project aimed to reveal a new facet of the biodiversity of coral reefs by shedding light on the complex links between genomes, transcriptomes, metabolomes, organisms, and ecosystem function in coral reefs and to provide a reference of the biological state of modern coral reefs in the Anthropocene. During its 2-year voyage, the Tara Pacific expedition sampled coral ecosystems from 32 islands across the Pacific Ocean and ocean surface waters at 249 locations, resulting in the collection of nearly 58,000 samples using various approaches (Table 1, Fig. 2). At each reef site, we systematically sampled two species of scleractinian corals, one hydrocoral, and two species of fish together with water and aerosols and environmental context data obtained from taxonomic registries, gazetteers, almanacs, climatologies, operational biogeochemical models, and satellite observations (Fig. 3)^4^. Following major advances in the field of marine biology made by the previous Tara expeditions like Tara Oceans^5^, the Tara Pacific program is now giving new insights into the diversity and plasticity of coral reef ecosystems. Here, we present major aspects of what we have learned from this unique and incomparable experience. We discuss these achievements on behalf of the Tara Pacific consortium and provide recognition of all the participants involved in the Acknowledgements.

**Table 1:** Number of different types of samples for the different targeted analyses

	Sequencing	Imaging	Phenotypes	Biogeochemistry
Fish	4236	1057	NA	NA
Coral	13421	12000	7797	NA
Coral core	NA	NA	NA	92
Water	NA	NA	NA	2059
Plankton	6782	3417	NA	944
Sediment	351	NA	NA	NA
Aerosol	1300	1323	NA	NA

*NA* not available.

**Fig. 2:**
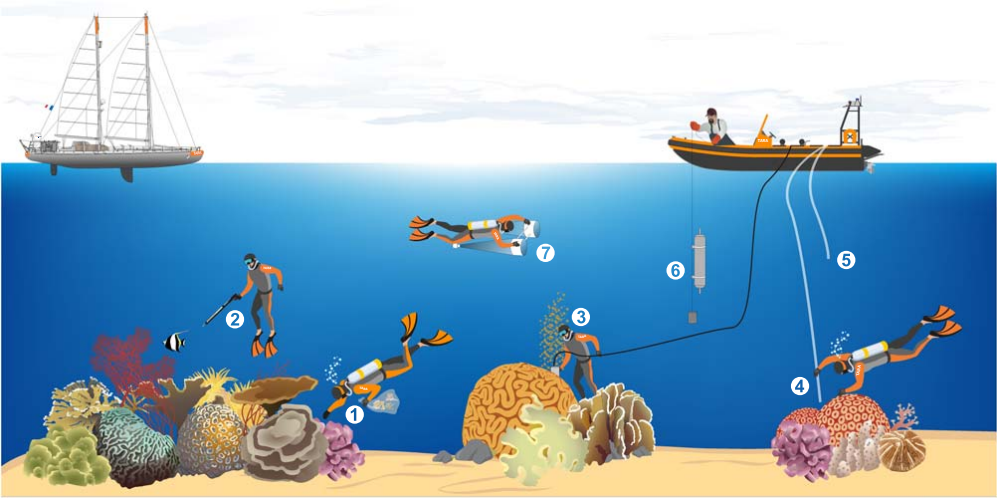
Schematic overview of the various sampling methods conducted during the Tara Pacific expedition while sampling on coral systems. (1) coral collection, (2) fish collection, (3) coral core sampling, (4) water collection at the surface of coral colonies, (5) superficial water collection, (6) Niskin water collection, and (7) plankton collection. Reproduced with permission from ref. ^4^.

**Fig. 3:**
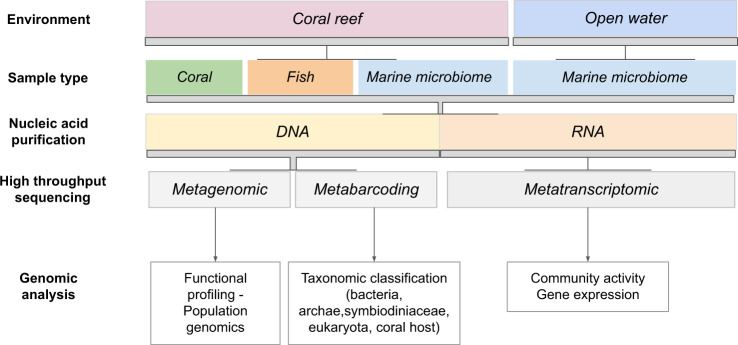
Overview of genomic analysis strategies deployed for the “omic” analysis of Tara Pacific samples. Reproduced with permission from ref. ^6^.

## Experimental design and organization

The first aspect is related to experimental design and organization. The baseline we developed, which used an efficient sample collection and processing strategy to meet numerous research demands and provide environmental context^4^, was instrumental in organizing and classifying the 3.8 million environmental data points collected across 74 metadata sources. In parallel, we set up a workflow for multi-omics data generation from sample handling to nucleotide sequence data generation and deposition^6^. This workflow makes it possible to manage what we think is the largest environmental science sequencing effort ever conducted to date, with approximately 102 Terabytes of metabarcode with different primers, metagenomes, and metatranscriptomes, as well as more than 5000 metabolomic profiles. Metabolomes were described by applying a wide-ranging analytical approach using both liquid chromatography–high-resolution mass spectrometry (for the lipidome) and nuclear magnetic resonance imaging (for the hydrophilic component) analyses to assess and annotate a broad range of the metabolome of three coral holobionts (Reddy et al., in prep.). This unique multidimensional framework also includes a large number of concomitant metadata collected side-by-side, all now publicly available for a larger audience.

### High-resolution dataset

The second aspect is linked to the description of the different ‘omics’ data sets. As such, in the framework of the Tara Pacific expedition, we assembled two coral genomes, *Porites lobata* and *Pocillopora meandrina*, with vastly improved contiguity that allowed us to study the functional organization of these genomes. We annotated their gene catalog and reported a relatively higher gene number than that found in other public coral genome sequences: 43,000 and 32,000 genes, respectively, which may be explained by a high number of tandemly duplicated genes^7^. Overall, the metabolomic analysis of the host was significantly distinct at higher taxonomic levels (at the genus level) but not within species (intraspecific). Instead, a large majority of the observed metabolomic variability within the host was explained by differences in biogeography (Reddy et al., in review).

We also demonstrate a very large richness of reef microbiota compared to other environments. If this diversity, highlighted in three Cnidaria and two fish, is applied to the number of fish and Cnidaria in the Pacific Ocean, then this diversity alone is equal to the total prokaryotic diversity currently estimated for the whole of the Earth, which suggests that the world’s microbial biodiversity is largely underestimated^8^. Microbial communities varied among and within the three animal biomes (coral, fish, and plankton) geographically. These microbiomes were species-specific and differed from those of the planktonic communities. Surprisingly, these microbiomes did not follow the well-known diversity gradients seen for corals^8^.

Within the coral microbiota, *Endozoicomonadaceae*, a globally distributed bacterial family, has been identified as a key bacterial symbiont of corals. A specific analysis of this taxon has now shown that the same clades are found across the Pacific Ocean but are host-specific at the species level and may harbor different specific functions^9^. We also show that within a coral genus, *Endozoicomonadaceae* biogeography is driven by the host rather than the environment^9^. Then, a survey of the viral compartment of the coral holobiont found heritable integrations of multiple *Dinornavirus* (a dinoflagellate-infecting non-retroviral RNA virus) endogenous viral element genes in *Symbiodiniaceae* scaffolds (especially that of the genus *Symbiodinium*) from within the cnidarian metagenomes. Such a result suggests widespread and recurrent or ancestral integration and conservation of these endogenous viral elements, which might have a role in reef health, for example, as an antiviral mechanism^10^.

## Unique outcomes

The third aspect is related to the unique outcomes beginning to emerge from analyses combining multi-omics and environmental data. The 32 archipelagos surveyed made for formidable natural laboratories and offered a wide range of environmental conditions in terms of temperature, acidification, and reef health state, making it possible to study the relationships between environmental and genetic parameters at large spatial scales. As an example, we provide evidence of high host–photosymbiont fidelity across environments in *Pocillopora* corals, with coral and microalgal gene expression profiles responding to different drivers^11^. Differences in the photosymbiotic association had only weak impacts on host gene expression, which was more strongly correlated with the historical thermal environment. Conversely, photosymbiont gene expression was largely driven by microalgal lineage. Overall, these results reveal a three-tiered strategy of heat resistance in *Pocillopora* underpinned by host–photosymbiont specificity, host transcriptomic plasticity, and differential photosymbiotic association under extreme warming. Utilizing the same samples, we analyzed the genetic diversity of the three coral genera in relation to climatic and environmental proxies^12^, showing that the impact of environments on evolutionary trajectories is species-specific. *Porites, Pocillopora*, and *Millepora* show different strategies: while the first has a resilient physiology with low biogeographical structuring, the other two corals show a stronger genomic imprint dependent on the environment, suggesting they are adapted to a narrower set of reef niches.

These different adaptative strategies in corals were confirmed by the study of stress markers (Porro et al., in review) and telomere length variation^13^. Porro et al. (in review) showed that *Porites* spp. has a stable phenotype within host lineages, while *Pocillopora* spp. have more diverse phenotypes structured by the geography and the environment, strategies which could be characterized as “the Oak and the Reed strategies”. A similar pattern was observed by measuring the telomere DNA length on 1029 colonies of the three coral genera with the telomere DNA lengths of the short-lived, more stress-sensitive *Pocillopora* spp. colonies being largely determined by seasonal temperature variation, whereas those of the long-lived, more stress-resistant *Porites* spp. colonies were insensitive to seasonal patterns but rather influenced by past thermal anomalies^13^.

Finally, we integrated the analysis of coral skeletal cores, which, like the trunk of a tree, retain the signature of events that prevailed at the time of their deposition (Canesi et al., in review). This property has made it possible to study the impact of different environmental conditions on the control of calcification and, therefore, on coral and reef growth. Throughout the Pacific Ocean, we show that the skeletal properties and calcifying fluid chemistry of *Porites* are primarily driven by the gradient in temperature. This unique comparative study of the massive coral *Porites* and *Diploastrea* at the Pacific Ocean basin scale demonstrated that the biological regulation of calcification parameters in their internal fluids in response to a temperature gradient is taxon-dependent.

## Outlook

The Tara Pacific expedition and program are unique and incomparable and will provide several years’ worth of material for large-scale analyses of coral ecosystem diversity. This program is unique in that samples were collected following the same protocol across multiple locations and years, with corals being screened in an identical manner at each site, rendering them fully comparable and linking their physiology to a large set of in situ and historical environmental data. The Tara Pacific expedition and program is not only the largest genotyping study conducted on a marine system, but it also represents a significant and large effort towards structuring and managing ecosystem-scale data to make them ‘open access’ and available to a broader community. By presenting summaries of our preliminary results above, we demonstrate the potential of such datasets for addressing major questions in the field of coral research. Answering these questions will lead to a better understanding of the conservation issues facing this unique ecosystem. It is our hope that the open-access data from the Tara Pacific expedition will provide an avenue for addressing these outstanding questions in coral reef research.
